# Alpha lipoic acid with pulsed radiofrequency in treatment of chronic lumbosacral radicular pain: A prospective, randomized study

**DOI:** 10.1097/MD.0000000000026344

**Published:** 2021-06-18

**Authors:** Khaled A. Abdelrahman, Abdelrady S. Ibrahim, Ayman M. Osman, Mohamed G. Aly, Abdelhady S. Ali, Waleed S. Farrag

**Affiliations:** Department of Anesthesia, ICU and Pain Management, Assiut University Hospital, Faculty of Medicine, Assiut, Egypt.

**Keywords:** alpha lipoic acid, lumbosacral radicular pain, oswestry disability index, pulsed radiofrequency

## Abstract

**Background:**

The effect of adding alpha lipoic acid (ALA) to pulsed radiofrequency (PRF) for treatment of lumbar-sacral pain was evaluated.

**Objective:**

to evaluate the effect of using ALA as an adjuvant therapy with PRF for treatment of chronic lumbosacral radicular pain caused by herniated disc.

**Methods:**

One hundred twenty patients with lumbo-sacral radicular pain allocated into 2 groups. Group I: treated with PRF at 42°C for 120 seconds. Group II: treated as in group I, plus oral ALA 600 mg (Thiotacid 600 mg, EVA PHARMA, Egypt) three times per day (1800 mg/day) for 3 weeks then 600 mg once daily for 2 weeks. The lumbo-sacral radicular pain evaluated using the numerical rating pain score and Oswestry Disability Index.

**Results:**

Success rate was significantly higher in group II at 3 and 6 months after intervention. The median values of the numerical rating pain score and the Oswestry Disability Index were significantly lower in group II with no significant difference in Epworth Sleepiness Scale. No major complications were reported in both groups.

**Conclusion:**

The current study supports the use of ALA with PRF on the dorsal root ganglion for treating lumbosacral radicular pain.

## 1 Introduction

Chronic lumbosacral radicular pain (LSRP) is primarily a neuropathic pain condition; its annual prevalence in the general population varies from 9.9% to 25%, meaning that it is the most common neuropathic pain.^[1]^ Patients who are suffering from this condition commonly experience severe pain, functional disability and psychological difficulties.^[2,3]^ Neuropathic pain characterized by peripheral and central sensitization of pain pathways with neural hyper-excitability. Therefore, LSRP can be regarded and treated as a common example of neuropathic pain.^[4]^

The main cause of LSRP is nerve root compression by herniated disc, ligaments bulge as a part of the degenerative process, and facet joint hypertrophy or the effect of epidural adhesion after spine surgeries.^[5,6]^ Nerve root compression can increase oxidative stress, which may lead to axonal degeneration and myelin degradation. Reactive oxygen species enhance synthesis of inflammatory cytokines and other inflammatory mediators that play an important role in inflammatory mechanism and the loss of axonal electrical conductivity.^[7,8]^

In the early 1950s, Reed and coworkers extracted alpha lipoic acid (ALA) from bovine liver,^[9]^ And since that date, ALA has been used in a variety of medical conditions, as cardiovascular diseases^[10]^ and diabetes mellitus.^[11]^ ALA is a multifunctional antioxidant and is effective in improving symptoms in diseases with an underlying oxidative stress elements.^[12]^ ALA reduces lipopolysaccharide-induced inflammatory responses in human monocytic cells.^[13]^ It also mediates cyclooxygenase-2^[14]^ and inducible nitric oxide synthase^[15]^ during inflammatory conditions with oxidative stress.

Several studies reporting the beneficial results of single pulsed radiofrequency (PRF) treatment or PRF in combination with conventional radiofrequency for the treatment of chronic LSRP have been published.^[16,17]^ ALA commonly used for treatment of some neuropathic pain and peripheral nerve injuries because it has antioxidant and free radical scavenging properties.^[18,19]^ It is believed to have a beneficial role in improving nerve conduction, and reducing pain and numbness and paresthesias in diabetic neuropathy.^[20]^

We hypothesized that ALA could reduce the progression of the nerve pathology via its anti-inflammatory action in the affected nerve. Therefore, in this study, we evaluated the effect of using ALA as an adjuvant therapy with PRF of the affected dorsal root ganglion (DRG) for treatment of chronic LSRP caused by herniated disc. Our primary outcome was assessment of the degree of pain relief and the occurrence of any complications was the secondary outcome.

## 2 Materials and methods

### 2.1 Study design

The study was prospective, randomized, and open label started in August 2013 and was completed in March 2017 at Pain Unit, Department of Anesthesia, ICU and Chronic Pain Management, Assiut University Hospital.

### 2.2 Ethical consideration

All patients were informed verbally and in writing about the study, and they signed a detailed written informed consent about the procedure and the nature of the study after local ethical committee approval and registration in ClinialTrials.gov ID: NCT03428139.

### 2.3 Eligibility criteria

Inclusion criteria included patients over 18 years with severe chronic LSRP and paresthesia in the affected dermatome of more than 6 months duration caused by herniated disc. Their pain intensity was ≥7 on the numerical rating scale^[21]^ and not responding to medical and conservative treatment such as physiotherapy, exercise therapy, analgesic medications or epidural injections. The diagnosis of nerve root pain was made based on reproduction of pain with known reliable and valid nerve root pain provocation procedures and neurologic assessment of sensation to pin prick, deep tendon reflexes and motor strength. Magnetic resonance imaging (MRI) was obtained in all patients using 1.5T MRI scanner (Magentom Avanto, Siemens Healthcare), the diagnosis of disc protrusion was confirmed, and electromyography was done for documentation of nerve root dysfunction.

Exclusion criteria included patients with history of previous spine surgery or indications for surgical intervention as significant severe and progressive motor deficits, cauda equina syndrome. Patients were excluded if they have some Other medical conditions as advanced malignancy, known concurrent neurological or neurodegenerative disease, including those with impaired neurotransmission e.g. myasthenia gravis, multiple sclerosis, spinal cord injury, pregnancy and nursing, active psychiatric or mental conditions, or those who takes other drugs with antioxidant effects such as vitamin A, vitamin C, vitamin E.

Patients with contraindication to the technique itself were also excluded as coagulopathy, allergy/sensitivity to lidocaine anesthetic or/ and non-ionic contrast media.

### 2.4 Randomization

Sequentially allocation was the type of randomization that we used to allocate patients in 2 different groups. Patients were enrolled based on sequentially numbered, according to the order in which they commence treatment at the clinic by a software program. A secretary of the pain unit was responsible to enroll and register patients in an appropriate group.

### 2.5 Study groups

One hundred twenty patients were sequentially allocated to be in one of 2 groups (60 in each). Group I: Each patient in this group treated with PRF on the affected DRG at 42°C for 120 seconds.^[22]^ Group II: Each patient in this group treated with PRF as in group I plus oral ALA 600 mg (Thiotacid 600 mg, EVA PHARMA, Egypt) three times per day (1800 mg/day) for 3 weeks then the dose was reduced to 600 mg once daily for 2 weeks. Studies showed that a dose of up to 1800 mg of ALA for 3 weeks are safe,^[23]^ after that the dose should be reduced to avoid oxidative damage of chronic high dose.^[24,25]^

### 2.6 Pulsed radiofrequency procedure

The patient placed in the prone position with a pillow under the lower abdomen to provide an easy approach to the intervertebral foramen. Under complete aseptic condition, 2 ml of 1% lidocaine (Lidocaine HCl, Hospira, Inc., Lake Forest, USA) used for local anesthesia of the skin prior to the placement of the RF electrode (22-G, 10 cm needle, with a curved 10 mm active tip, Neurotherm) under C-arm fluoroscopic control. In the lumbar segments, the electrode was positioned near to the DRG, which typically corresponded to the dorsal-cranial quadrant of the intervertebral foramen on lateral view, and on anteroposterior view, the tip was located midway in the pedicle column. The electrode advancement was depending on the stimulation criteria below. Once the electrode was in a correct position, the stylet then replaced by the radiofrequency probe. PRF current applied for 20 ms, at 2 Hz, for 120 seconds. The maximum target temperature was 42°C (Neurotherm 1100).

The final positional confirmed by the following methods:

1. Sensory stimulation (50 Hz) threshold under 0.6 volts, which made paresthesia in the usual distribution of radicular pain.
2. Motor stimulation caused no muscle contraction at corresponding myotome below 2 volts to ensure that the probe was far away from ventral root cell and so close to DRG.

Weak muscle contraction above 2 volts was accepted to be targeted treatment. PRF current applied for 20 ms, at 2 Hz, for 120 second. The maximum target temperature was 42°C (Neurotherm 1100).

### 2.7 Follow-up

All patients were admitted after the procedure into a ward for at least 2 hours with, recording of any adverse effects or complications. Blood pressure and pulse oximetry continuously monitored using Patient Monitor Umec12, Mindray, China. Any complaints such as pain, vomiting, or leg weakness was reported. Vomiting was treated by ondansetron 4 mg through intravenous route, whereas pain in the back or leg managed by reassurance and non-steroidal analgesic drugs after excluding serious events. Before discharge, each patient received the necessary instructions and contact numbers.

### 2.8 Outcome measures

Patients’ responses to the treatment were followed up on subsequent visits to the pain clinic at 3 and 6 months for the degree of radicular pain relief and the occurrence of any complications. If a patient was unable to attend for follow-up, a detailed telephone interview was conducted by a pain physician blinded to the patient group.

Numerical rating score: The radicular pain was evaluated using standard 11-point scaled with 0 being no pain, and 10 representing the worst pain imaginable. Pain relief was considered a success if the treatment resulted in a numerical rating scale <4 points for at least 12 weeks.

Oswestry Disability Index: Lumbo-sacral pain was assessed by the Oswestry Disability Index (range 0–100), for the 10 items of the score, each mean score (range; 0, normal to 5, impossible) was recorded separately and compared.^[26]^

Satisfaction score: The patients’ satisfaction grades, which were presented as very good (grade I), good (grade II), fair (grade III), bad (grade IV), or very bad (grade V), were also evaluated by questionnaire. “Satisfied” was defined as a response of very good or good in the questionnaire.^[27]^

The Epworth Sleepiness Scale 0 = would never doze, 1 = slight chance of dozing, 2 = moderate chance of dozing and 3 = high chance of dozing. Score Results: 1 to 6 less chance to sleep, 7 to 8 average sleep and 9 and up Very sleepy.^[28]^

### 2.9 Sample size

We relied on the previous study,^[29]^ which was conducted in the same setting, to calculate the sample size. This was based on an 80% power of the study and a 95% confidence interval. We included 60 patients in each group to achieve these parameters.

### 2.10 Statistical analysis

All data analyzed by using SPSS software (version 20). Per-protocol population was analyzed and all descriptive statistics (quantitative parameters) of the study were done in the form of mean (confidence interval), and median (minimum-maximum), absolute and relative frequencies were used to report qualitative variables. In categorical data, Chi-Squared test was used and Mann–Whitney test was used for non-parametric data. *P* values were considered statistically significant if <.05 (confidence interval 95%).

## 3 Results

**Figure 1:**
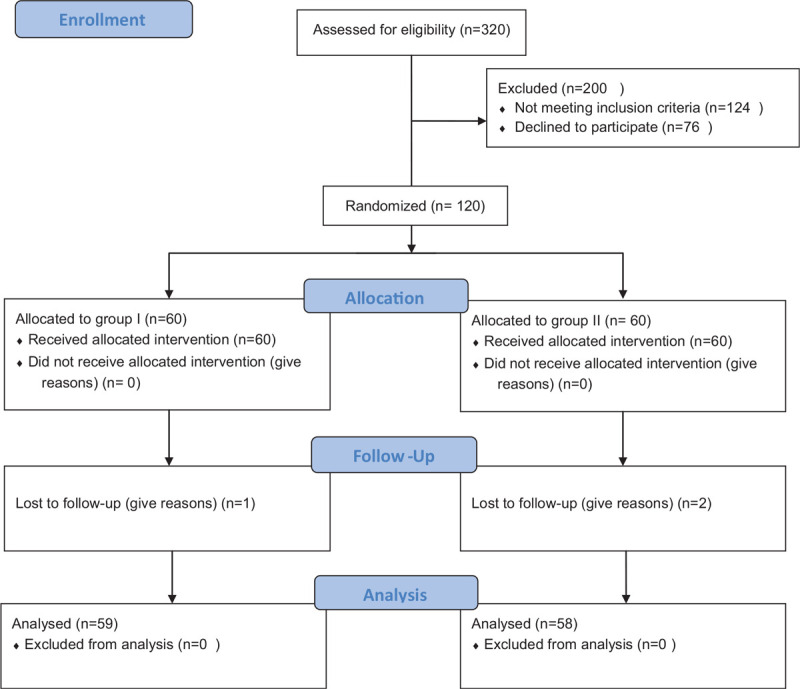
Consort Flow Diagram.

Three hundred twenty patients assessed for eligibility; 200 were excluded because they did not meet the inclusion criteria or refused to participate in the study. The remaining 120 patients were allocated and randomly divided into 2 equal groups. Three patients were excluded from statistical analysis due to lost to follow-up. The Group I included 59 patients and group II included 58 patients as shown in the consort flow diagram (Fig. 1). The patients in both groups were comparable as regard their demographic and clinical data as shown in Table 1.

**Table 1:** Demographic and clinical data in the study groups.

	Group I (N = 59)	Group II (N = 58)
Age (yr)	46.95 ± 13.94	54.0 ± 10.30
Sex		
Male	37 (62.7%)	41 (70.7%)
Female	22 (37.3%)	17 (29.3%)
Weight (kg)	76.76 ± 7.45	78.80 ± 8.73
Height (cm)	176.6 ± 9.7	175.0 ± 8.6
Medical History:		
D.M	9 (15.3%)	7 (12.1%)
Hypertension	14 (23.7%)	16 (27.6%)
Nerve root affected:		
L5	23 (38.9%)	21 (36.2%)
L4	14 (23.8%)	18 (31.1%)
L3	2 (3.4%)	2 (3.5%)
L2	2 (3.4%)	1 (1.7%)
L4 / L5	16 (27.1%)	13 (22.4%)
L3/ L4	2 (3.4%)	3 (5.1%)

Pain score (Table 2): Before the treatment, numerical rating scale did not show a significant difference between the studied groups and its median value recorded an average score 8 in both groups (*P* = .07). There were statistically significant differences in patients who received ALA, compared to patients who did not receive after 3 and 6 months (*P* values = .005 and .011 respectively). The median value (minimum-maximum) of pain score for patients who did not receive an ALA was 4 (1–6) after 3 months and it was 4 (2–6) after 6 months, whereas in the group who received the drug it was 3 (0–6) and 3 (2–6) respectively.

**Table 2:** Numerical rating pain score, Disability score and epworth sleepiness scale in the study groups.

	Group I (N = 59)	Group II (N = 58)	*P* value
Numerical rating pain score			
Before the procedure	8.0 (6–9)	8.0 (7–9)	.071
After the procedure:			
3 mo	4.0 (1–6)	3.0 (0–6)	.005^∗^
6 mo	4.0 (2–6)	3.0 (2–6)	.011^∗^
Oswestry Low Back Pain Disability score			
Before the procedure	35.0 (15–60)	30 (20–60)	.349
After the procedure:			
3 months	17.0 (5–40)	10 (5–35)	.097
6 months	15 (10–35)	12 (10–30)	.048^∗^
Epworth Sleepiness Scale			
Before the procedure	6 (5–7)	6 (5–7)	.93
After the procedure:			
3 months	9 (7–9)	9 (6–10)	.07
6 months	9 (7–9)	9 (7–10)	.13

Disability score (Table 2): The median value of the Oswestry Low Back Pain Disability for all patients before starting the study was comparable (*P* = .349). After 3 and 6 months, for patients who did not receive an ALA, the median value went down from 35 (15–60) before the procedure to 17 (5–40) and 15 (10–35) respectively, whereas in patients who received the drugs disability index declined from 30 (20–60) before the study to 10 (5–35) and 12 (10–30) respectively. The P value between the 2 groups was 0.097 after 3 months (statistically insignificant) and 0.048 after 6 months (statistically significant).

The Epworth Sleepiness Scores (Table 2): The median values of Epworth Sleepiness Scale after 3 months between group I and group II were 9 (7–9) and 9 (6–10) respectively; *P* value was .07 (statistically insignificant). After 6 months, sleep scores of these groups become 9 (7–9) and 9 (7–10) respectively; *P* value was .132 (statistical insignificant).

The success rate (Table 3): The percentage of patients who showed successful treatment was significantly higher (<0.001) in group II during the follow up period at 3 and 6 months. At 3 months 46 patients in group I [78.0% (CI 67%–89%)] and 53 patients in group II [91.4% (CI 84%–99%)] had numerical rating scale < 4 indicating successful outcome from the procedure. At 6 months 38 patients in group I [64.4% (CI 52%–77%)] and 47 patients in group II [81.03% (CI 71%–91%)] showed successful outcome.

**Table 3:** Success rate after treatment in the study groups.

	Group I (N = 59)	Group II (N = 58)	*P* value
3 mo	46 (78.0%) (CI 67%-89%)	53 (91.4%) (CI 84%-99%)	<.001^∗^
6 mo	38 (64.4%) (CI 52%-77%)	47 (81.03%) (CI 71%-91%)	<.001^∗^

The patients’ satisfaction grades (Table 4): The overall satisfaction score was significantly better (*P* < .001) in group II than group I at 3 and 6 months after the procedure. Ten patients (10.7%) at 3 months and 20 patients (34.5%) at 6 months in group II had grade I satisfaction score versus 0% in group I.

**Table 4:** Patients’ satisfaction grades in the study groups.

	Patient satisfaction	After treatment
Grades	3 mo	6 mo
Group I (N = 59)		
Grade I	0 (0%)	0 (0%)
Grade II	4 (6.8%)	2 (3.4%)
Grade III	14 (23.7%)	29 (49.2%)
Grade IV	41 (69.5%)	28 (47.5%)
Grade V	0 (0%)	0 (0%)
Group II (N = 58)		
Grade I	10 (17.2%)	20 (34.5%)
Grade II	14 (24.1%)	24 (41.4%)
Grade III	12 (20.7%)	14 (24.1%)
Grade IV	22 (37.9%)	0 (0%)
Grade V	0 (0%)	0 (0%)
P value	<0.001^∗^	<0.001^∗^

Side effects: There were no neurological deficits such as motor loss or hypersensitivity in either of the groups. Some cases in both groups complained of burning dermatomal pain and leg weakness which relieved after a short period. Most Patients who received ALA reported gastric pain and 3 cases of them did not complete the full dose of treatment, but they were not excluded from the study because they missed the last days’ doses (3, 2 and 2 doses) because they did not fully understand the instructions (the missed doses were less than 4% of the total dosage scheduled for these patients, that is why they were considered insignificant).

## 4 Discussion

The results in our study showed that ALA could lead to better pain relief and function ability with patient satisfaction and increase the success rate of treatment up to 6 months, when used as an adjuvant treatment with DRG pulsed radiofrequency in the people who were suffering from lumbo-sacral radicular pain resulted from herniated disc.

Lumbo-sacral radicular pain commonly affects sciatic nerve and lower lumbar nerve roots and the lifetime incidence of this condition is estimated to be between 13% and 40% potential to become chronic and intractable, with major socioeconomic implications.^[30]^ It could be proposed that radicular pain in sciatic nerve roots arises from a complex interaction of inflammatory, immune, and pressure-related elements.^[31]^ The study of Das et al, 2018 supported the concept that chronic radicular pain is a centrally mediated neuroimmune phenomenon and the mechanism of action of DRG PRF treatment is immunomodulatory.^[32]^

Van Zundert et al, 2007 concluded that the PRF treatment adjacent to the cervical DRG was advocated and reported to show significant pain relief compared with placebo at 3 months follow-up for the treatment of cervical radicular pain,^[33]^ and it has been used to treat many other painful conditions such as shoulder pain and occipital neuralgia.^[34]^

ALA has been described as a potent biological antioxidant, a detoxification agent, and a diabetes medicine; used mainly for treating diabetic neuropathy conditions. It has been also used to improve age-associated cardiovascular, cognitive, and neuromuscular deficits, and has been implicated as a modulator of various inflammatory signaling pathways.^[35]^

ALA has been well tolerated with minimal and mild side effects usually occur only at doses greater than 600 mg. Possible side effects include nausea, gastric pain, vomiting, and vertigo. In addition, in IV administration, local pain during infusion and redness are common.^[36]^ Various long term studies using both oral and IV administration have not resulted in any significant adverse effects when compared to placebo.^[37]^ There have been some cases of spontaneous hypoglycemia due to the development of insulin autoimmune syndrome associated with the administration of ALA.^[38]^

The meta-analysis of the study of Ziegler et al, 2004; provided evidence that treatment with ALA (600 mg per day iv.) over 3 weeks is safe and significantly improves both positive neuropathic symptoms and neuropathic deficits to a clinically meaningful degree in diabetic patients with symptomatic polyneuropathy.^[39]^

Battisti et al, 2013; reported a statistically significant reduction of pain and functional disabilities in patients who received ALA. Pain improved after a few weeks of treatment and this improvement was statistically and clinically significant. Four patients could not continue the treatment due to severe gastric pain. They concluded that treatment with ALA and Superoxide dismutase (SOD) has a great benefit to improve functional disabilities and decrease the need for analgesics in chronic LSRP patients.^[40]^

Ranieri et al, 2009; cited that 6 weeks of oral treatment with ALA and gamma-linolenic acid (GLA) in combination with rehabilitation therapy alleviated neuropathic symptoms and neurological deficits in patients who are suffering from radicular neuropathy.^[18]^

In our study, ALA was added to PRF in treatment of chronic LSRP. Nerve root compression is known to increase oxidative stress. Reactive oxygen species enhance synthesis of inflammatory cytokines and other inflammatory mediators.^[7]^ We used ALA at a dose of 1800 mg per day for 3 weeks then 600 mg per day for another 2 weeks, however, the effect of ALA continued up to 6 months. ALA is known to have both free radical scavenging and nutritional effects.^[18,41]^ We assume that these effects allowed the compressed nerves to improve its function better than the use of PRF alone. The clinical success of ALA in improving pain, disability and satisfaction when added to PRF could at least be attributed partially to its ability to improve the nerve function and inflammation via the above-mentioned mechanisms.

PRF of the DRG showed an immunomodulating effect, causing a shift in the immune system balance with a decreased production of pro-inflammatory cytokines such as TNF-α and IL-1 and a raised anti-inflammatory status.^[42]^ PRF treatment modulates lymphocytes and neuroinflammatory markers in chronic radicular pain,^[32]^ Human DRG PRF treatment modulates cerebrospinal fluid lymphocytes and neuroinflammatory markers in chronic radicular pain. cited). ALA also exert multi-level immunomodulatory functions,^[43,44]^ since both ALA and PRF to DRG share immunomodulatory effects we may suggest ALA may enhance the immunomodulatory effects of PRF on the DRG.

However, many patients experienced improved disability and pain scores after treatment but were not satisfied. This could be attributed, in part, to not fulfilling their expectations. In addition, the chronicity of the problem may have its psychological impacts. On the other hand, no patient in either groups reported (very bad) in the satisfaction score at 3 and 6 months, and no patient in group II reported (bad) at 6 months, this could be attributed to the partial improvement of pain and disability. Anyhow, satisfaction scores are multifactorial, and sometimes are difficult to explain as patients expectations, psychological status, doctors behavior, nursing and staff care, life style and many other variables can possibly affect patients satisfaction.^[45]^

The present study has some limitations. First, because the aim of the study was to provide evidence of the benefit of ALA as adjuvant therapy with no placebo medication used, participants and medical stuff were not blinded. Short-term period of the study is another limitation and we recommend further studies with follow up period up to 12 month.

## 5 Conclusion

The current study supports the use of ALA with PRF on the DRG for treating LSRP resulting from a herniated disc. The pain score significantly decreased at 3 and 6 months, and improvement in disability score at 6 month without any significant side effects. We postulate that oral supplementation of ALA helps in reducing progression of the nerve pathology via its anti-inflammatory action in the affected nerve.

## Acknowledgments

We thank all our participants and staff of Pain Unit, Assiut University hospital, without whose efforts none of this work would have been possible.

## Author contributions

**Conceptualization:** Ayman Mamdouh Osman.

**Data curation:** Khaled A. Abdelrahman, Mohammed Galal Aly.

**Formal analysis**: Khaled A. Abdelrahman, Abdelhady S. Ali, Waleed S. Farrag.

**Investigation:** Ayman Mamdouh Osman.

**Methodology:** Khaled A. Abdelrahman, Waleed S. Farrag.

**Project administration:** Abdelrady Shehata Ibrahim.

**Supervision:** Mohammed Galal Aly, Waleed S. Farrag.

**Validation:** Abdelhady S. Ali.

**Writing – original draft:** Abdelrady Shehata Ibrahim, Abdelhady S. Ali.

**Writing – review & editing:** Waleed S. Farrag.
